# Analysis of risk factors of multiorgan failure after pericardiectomy for constrictive pericarditis

**DOI:** 10.1186/s13019-022-02007-1

**Published:** 2022-09-30

**Authors:** Jing-bin Huang, Zhao-ke Wen, Jian-rong Yang, Jun-jun Li, Min Li, Chang-chao Lu, Da-ying Liang, Cheng-xin Wei

**Affiliations:** 1 Department of Cardiothoracic Surgery, The People’s Hospital of Guangxi Zhuang Autonomous Region, and Guangxi Academy of Medical Sciences, 6 Taoyuan Road, Nanning, 530021 Guangxi China; 2 Department of Cardiothoracic Surgery, Ruikang Hospital Affiliated to Guangxi University of Chinese Medicine, 10 Huadong Road, Nanning, 530011 Guangxi China; 3 Department of Cardiothoracic Surgery, The People’s Hospital of Liuzhou City, 8 Wenchang Road, Liuzhou, 545006 Guangxi China

**Keywords:** Incomplete pericardial dissection, Fluid overload, multiorgan failure, Pericardiectomy

## Abstract

**Background:**

We aimed to investigate risk factors of multiorgan failure following pericardiectomy.

**Methods:**

This was a retrospective study of patients undergoing pericardiectomy between January 1994 and May 2021 at three hospitals.

**Results:**

826 patients were included in the study and divided into two groups: group with multiorgan failure (n = 86) and group without multiorgan failure (n = 740). There were 86 patients with multiorgan failure (86/826, 10.4%). There were 66 operative deaths (66/826, 8.0%). The causes of operative deaths were multiorgan failure, including cardiogenic shock + AKI + ventricular fibrillation (13/66), cardiogenic shock + AKI (35/66), cardiogenic shock + AKI + hepatic failure + septicemia (8/66), cardiogenic shock + AKI + respiratory failure (10/66). Univariate and multivariate analyses showed the factors associated with multiorgan failure, including male (*P* = 0.006), time between symptoms and surgery (*P* < 0.001), thickness of pericardium (*P* < 0.001), intubation time (*P* < 0.001), ICU retention time (*P* < 0.001), hospitalized time postoperative (*P* < 0.001), preoperative central venous pressure (*P* < 0.001), postoperative central venous pressure (*P* < 0.001), D0 fluid balance (*P* < 0.001), D2 fluid balance (*P* < 0.001), postoperative chest drainage (*P* < 0.001), preoperative LVEDD(*P* < 0.001), postoperative LVEDD (*P* < 0.001), surgical duration (*P* < 0.001), bleeding during operation (*P* < 0.001), serum creatinine 24 h after surgery (*P* = 0.042), serum creatinine 48 h after surgery (*P* < 0.001), fresh-frozen plasma (*P* < 0.001), packed red cells (*P* < 0.001), blood lactate (*P* < 0.001).

**Conclusion:**

In our study, incomplete pericardial dissection, fluid overload, delayed diagnosis and treatment are associated with multiorgan failure following pericardiectomy.

## Background

Constrictive pericarditis is the results of chronic inflammation characterized by fibrous thickening and calcification of the pericardium that injuries diastolic filling, decreases cardiac output, and ultimately results in heart failure. While the operative mortality risk of pericardiectomy is still high and ranges between 5 and 20% [1, 2]. Determining the risk factors of multiorgan failure after pericardiectomy for constrictive pericarditis has clinical significance for the management of patients undergoing pericardiectomy. The objective of this study was to determine the risk factors of multiorgan failure following pericardiectomy.

## Patients and methods

### Design

This was a retrospective, observational cohort study of patients undergoing pericardiectomy between January 1994 and May 2021 at The People’s Hospital of Guangxi Zhuang Autonomous Region, Ruikang Hospital Affiliated to Guangxi University of Chinese Medicine, and The People’s Hospital of Liuzhou City. Medical records were reviewed.

#### Eligibility criteria

##### Inclusion criteria

Patients undergoing pericardiectomy between January 1994 and May 2021 at The People’s Hospital of Guangxi Zhuang Autonomous Region, Ruikang Hospital Affiliated to Guangxi University of Chinese Medicine, and The People’s Hospital of Liuzhou City.

##### Exclusion criteria

Patients with missing medical records.

#### Variables to be analyzed

Variables to be analyzed included clinical parameters (gender, age, weight before diuresis, weight after diuresis, cachexia, pulmonary tuberculosis, rheumatic heart disease, infective endocarditis, valvular heart disease, coronary heart disease, pleural effusion, thickened pericardium, pericardial effusion, pericardial calcification, serum creatinine, and central venous pressure), echocardiographic parameters (left ventricular end diastolic dimension, left ventricular ejection fractions, aortic insufficiency, mitral regurgitation, and tricuspid regurgitation), preoperative parameters (time between symptoms and surgery, thickness of pericardium, and NYHA class), postoperative parameters (mean intubation time, ICU retention time, hospitalized time after surgery, postoperative chest drainage, surgical duration, bleeding during operation, fresh-frozen plasma, packed red cells, and fluid balance on operation day, the first day following operation and the second day following operation), and therapeutic parameters (low cardiac output syndrome, acute renal injury, multiorgan failure, long-term intubation, empyema, hepatic failure, respiratory failure, ventricular fibrillation, use of inotropic medication, blood lactate, extracorporeal membrane oxygenation requirement, and death).

### Preoperative diuresis protocol

Hydrochlorothiazide tablet 25 mg bid, furosemide tablet 20 mg bid. Diuresis treatment last 7 to 30 days.

Low cardiac output syndrome: all patients were monitored with a pulmonary artery catheter in the operation room and intensive care unit, cardiac output and venous oxygen saturation of hemoglobin were continuously measured. Low cardiac output syndrome is defined by a cardiac index of less than 2.0 L/min/m^2^ in the operation room and intensive care unit. LCOS is characterized by clinical signs or symptoms including elevated blood lactate or rapid increase in blood lactate, decreased central venous oxygen saturation, increased arterial to central venous oxygen saturation difference, decreased urine output, increased peripheral skin temperature to core body temperature difference, and low echocardiographic Doppler-derived cardiac index, high inotrope requirement [3].

Postoperative LVEDD was measured by transthoracic echocardiography postoperatively 1 to 7 days in intensive care unit.

Perioperative death was defined as death within 30 days of the operation or during the same hospital admission.

Serum creatinine was used as the diagnostic standard of acute renal injury. According to Kidney Disease Improving Global Outcomes classification, if serum creatinine increases by ≥ 0.3 mg/dl (26.5 μmol/l) within 48 h, serum creatinine is 50% higher than the baseline within first seven days, or urine output is below 0.5 ml/kg/h for 6 h, the patient is considered to have acute renal injury [4].

Multiorgan failure is regarded as a continuous process of varying levels of organ failure rather than an all-or-none event [5]. To characterize multiorgan failure, six different organ systems are regarded as “key organs”: lungs, cardiovascular system, kidneys, liver, coagulation system, and central nervous system.

Hepatic failure is defined as “a severe liver injury, potentially reversible in nature and with onset of hepatic encephalopathy within 8 weeks of the first symptoms in the absence of pre-existing liver disease [6].

Respiratory failure is a condition in which the respiratory system fails in one or both of its gas exchange functions. It is defined by an arterial oxygen tension of ≤ 8.0 kPa (60 mmHg), an arterial carbon dioxide tension of ≥ 6.0 kPa (45 mmHg) or both [7].

### Diagnosis of constrictive pericarditis

The diagnosis of constrictive pericarditis was made on the basis of clinical manifestation, echocardiography, chest computed tomography scan, cardiac catheterization, surgery, and pathological criteria. Typical symptoms and signs are a prominent change in the x and y descent in jugular venous pulse, dyspnea upon exertion, palpitations, abdominal distension, as well as edema in the ankles or legs. Echocardiography and chest computed tomography scan revealed a severely thickened or calcified pericardium and cardiac catheterization revealed elevated end-diastolic pressure and the “square root sign” of right ventricular pressure tracing. Surgical and pathological findings were reviewed to confirm the preoperative diagnosis.

### Surgical technique

Pericardiectomy was performed via sternotomy, the pericardium was removed between the two phrenic nerves and from the great vessels to the basal aspect of the heart. The pericardium was palpated to identify a relatively soft and uncalcified area after median sternotomy, and the thymus removed laterally. Dissection was started at the base of the aorta, extended downwards to the lateral and posterior walls of the left ventricle, followed by the diaphragmatic pericardium. The pericardium over the right atrium and venae cava was resected last. If calcified plaques penetrating the epicardium were present, we left small “islands” of calcified pericardial tissue. Cardiopulmonary bypass was avoided during surgery except for concomitant valve replacement.

### Follow-up

All survivors discharged from hospital were monitored to the end date of the study. At the outpatient department, all patients were investigated with echocardiogram, electrocardiogram, and X-ray chest film, once every 3 to 12 months. At the last follow-up, the patients were contacted by telephone or micro-massage or interviewed directly at the outpatient department.

### Statistical analyses

Continuous variables are reported as means ± SE. Survival rates were estimated using the Kaplan–Meier method. The chi-square test, the Kruskal-Walls test or the Wilcoxon rank-sum test, as appropriate, to be used to evaluate relationships between the preoperative variables, and selected intraoperative and postoperative variables. The relationships with perioperative risk factors were assessed by means of contingency table methods and logistic regression analysis. *P* values less than 0.05 were considered to be statistically significant. All analyses were performed using IBM SPSS version 24.0 software (IBM SPSS Inc., USA).

Univariate logistic regressions with multiorgan failure as an outcome were analyzed first. Then, the Variance Inflation Factor was calculated to explore the independence of the selected variables. The results are listed in Table 5, and there is no evidence to show dependence among the selected factors. Therefore, the significant variables were entered into multiple logistic regressions without an interaction term, and the stepwise variable selection method was used to identify the potential risk factors of multiorgan failure**.**

### Ethics approval

The experiment protocol for involving humans was in accordance to Helsinki Statement and national guidelines and was approved by the Medical Ethics Committee of The People’s Hospital of Guangxi Zhuang Autonomous Region, Ruikang Hospital Affiliated to Guangxi University of Chinese Medicine, and The People’s Hospital of Liuzhou City, and They gave the authors approval to waive the need for patient consent for publishing data in the study about the patients.

## Results

### Characteristics of the population under study

During the study period, 829 patients underwent pericardiectomy; of them, 3 met the exclusion criteria, so a total of 826 patients were eligible and included in the study group.

### Preoperative and operative data

826 consecutive patients undergoing pericardiectomy for constrictive pericarditis were included in the study. The patients were divided into two groups: group with multiorgan failure (n = 86) and group without multiorgan failure (n = 740) (Tables 1, 2). In our study, time between symptoms and surgery (22.9 ± 4.1 vs. 6.7 ± 0.5 month, *P* < 0.001), thickness of pericardium (22.7 ± 0.6 vs. 19.8 ± 0.2 mm, *P* < 0.001), preoperative CVP (25.0 ± 0.4 vs. 19.3 ± 0.2 mmHg, *P* < 0.001) in group with multiorgan failure were significantly higher than those in group without multiorgan failure (Table 2).

**Table 1:** Preoperative characteristics of the patients (n = 826)

Variable	Value
Female/male, n	280/546
Age, years	53.9 ± 0.6 (range 17.0 to 73.0)
Weight before diuresis, kg	56.1 ± 0.4 (range 36.0 to 80.0)
Weight after diuresis, kg	53.8 ± 0.4 (range 34.0 to 75.0)
Time between symptoms and surgery, month	9.3 ± 0.9 (range 0.3 to 120.3)
BMI before diuresis, kg/m^2^	21.9 ± 0.1 (range 15.4 to 31.3)
BMI after diuresis, kg/m^2^	21.0 ± 0.1 (range 14.5 to 28.2)
NYHA class	
II, n	462 (55.9%)
III, n	347 (42.0%)
IV, n	17 (2.1%)
Cachexia, n	33 (4.0%)
Pulmonary tuberculosis, n	17 (2.1%)
Rheumatic heart disease, n	33 (4.0%)
Infective endocarditis, n	9 (1.1%)
Valvular heart disease, n	34 (4.1%)
Coronary heart disease, n	28 (3.4%)
Pleural effusion, n	74 (9.0%)
Preoperative LVEDD, mm	41.7 ± 0.2 (range 29.0 to 60.0)
Preoperative LVEF, %	62.5 ± 0.3 (range 51.0 to 77.0)
Aortic insufficiency, n	58 (7.0%)
Mitral regurgitation, n	70 (8.5%)
Preoperative tricuspid insufficiency, cm^2^	1.8 ± 0.1 (range 0.0 to 13.5)
Thickened pericardium, n	825 (99.9%)
Thickness of pericardium, mm	20.2 ± 0.3 (range 3.0 to 30.0)
Tuberculosis pericarditis, n	434 (52.5%)
Pericardial effusion, n	406 (49.2%)
Pericardial calcification, n	196 (23.7%)
Patients with CPB, n	76 (9.2%)

826 consecutive patients undergoing pericardiectomy for constrictive pericarditis were included in the study. BMI = weight/(height^2^), (kg/m^2^)

**Table 2:** Preoperative data

Variable	Group With multiorgan failure (n = 86)	Group without multiorgan failure (n = 740)	*P* value
Male, n (%)	(%)	(%)	
Age, years	53.0 ± 1.7	53.8 ± 0.5	0.618
Weight before diuresis	53.0 ± 1.0	56.3 ± 0.4	0.003
Weight after diuresis	51.1 ± 0.7	54.0 ± 0.3	0.002
Preoperative CVP, mmHg	25.0 ± 0.4	19.3 ± 0.2	< 0.001
Preoperative LVEDD, mm	39.6 ± 0.5	42.0 ± 0.2	< 0.001
Preoperative LVEF, %	63.5 ± 0.9	62.4 ± 0.2	0.137
Baseline serum creatinine, μmol/l	82.3 ± 2.9	79.4 ± 1.2	0.424
Height, cm	154.3 ± 1.2	160.7 ± 0.3	< 0.001
BMI before diuresis, kg/m^2^	22.3 ± 0.4	21.7 ± 0.1	0.136
BMI after diuresis, kg/m^2^	21.6 ± 0.3	20.9 ± 0.1	0.041
Time between symptoms and surgery, months	22.9 ± 4.1	6.7 ± 0.5	< 0.001
Thickness of pericardium, mm	22.7 ± 0.6	19.8 ± 0.2	< 0.001

In our study, time between symptoms and surgery (22.9 ± 4.1 vs. 6.7 ± 0.5 month, *P* < 0.001), thickness of pericardium (22.7 ± 0.6 vs. 19.8 ± 0.2 mm, *P* < 0.001), preoperative CVP (25.0 ± 0.4 vs. 19.3 ± 0.2 mmHg, *P* < 0.001) in group with multiorgan failure were significantly higher than those in group without multiorgan failure

#### Mortality

There were 66 operative deaths (66/826, 8.0%). The causes of operative deaths were multiorgan failure, including cardiogenic shock + AKI + ventricular fibrillation (13/66), cardiogenic shock + AKI (35/66), cardiogenic shock + AKI + hepatic failure + septicemia (8/66), cardiogenic shock + AKI + respiratory failure (10/66).

#### Resource utilization

##### Patient required extracorporeal membrane oxygenation

Fluid balance on operation day (D0) of group with multiorgan failure were significantly less negative than that of group without multiorgan failure (− 521.2 ± 52.0 ml vs. − 1185.8 ± 31.5 ml, *P* < 0.001). While fluid balance postoperative day D2 of group with multiorgan failure was significantly more negative than that of group without multiorgan failure (− 1465.4 ± 154.9 ml vs. − 506.2 ± 23.0 ml, *P* < 0.001). Use of adrenaline of group with multiorgan failure were significantly higher than that of group without multiorgan failure (100% vs. 25.9%, *P* < 0.001; 1.8 ± 0.02 vs. 0.02 ± 0.01 μg/kg/min, *P* < 0.001; respectively). Chest drainage (1483.6 ± 85.7 vs. 793.8 ± 17.8 ml, *P* < 0.001), and surgical duration (215.1 ± 6.1 vs. 179.7 ± 2.2 min, *P* < 0.001) of group with multiorgan failure were significantly more than those of group without multiorgan failure (Table 3).

**Table 3:** Operative data

Variable	Group with multiorgan failure (n = 86)	Group without multiorgan failure (n = 740)	*P* value
Intubation time, hours	147.0 ± 10.4	56.0 ± 2.0	< 0.001
ICU retention time, days	12.3 ± 1.1	4.5 ± 0.1	< 0.001
Hospitalized time postoperative, days	23.6 ± 3.1	14.8 ± 0.2	< 0.001
Postoperative CVP, mmHg	13.3 ± 0.2	11.5 ± 0.1	< 0.001
D0 fluid balance, ml	− 640.7 ± 52.0	− 1223.9 ± 31.6	< 0.001
D1 fluid balance, ml	− 510.0 ± 201.8	− 555.6 ± 31.6	0.693
D2 fluid balance, ml	− 1176.1 ± 131.6	− 478.9 ± 20.9	< 0.001
Chest drainage, ml	1483.6 ± 85.7	793.8 ± 17.8	< 0.001
Serum creatinine 24 h after surgery, μmol/l	107.7 ± 4.0	78.6 ± 0.8	< 0.001
Serum creatinine 48 h after surgery, μmol/l	167.3 ± 5.2	89.1 ± 1.1	< 0.001
Fresh-frozen plasma, ml	1439.0 ± 153.1	519.3 ± 20.1	< 0.001
Packed red cells, unit	1.0 ± 0.1	0.4 ± 0.1	< 0.001
Surgical duration, min	230.0 ± 5.7	174.1 ± 2.1	< 0.001
Adrenaline, %	100% (66/66)	25.9% (197/760)	< 0.001
Adrenaline,	1.8 ± 0.02	0.02 ± 0.01	< 0.001
Blood lactate,	12.5 ± 0.5	2.1 ± 0.1	< 0.001

Fluid balance on operation day (D0)of group with multiorgan failure were significantly less negative than that of group without multiorgan failure (− 521.2 ± 52.0 ml vs. − 1185.8 ± 31.5 ml, *P* < 0.001). While fluid balance postoperative day D2 of group with multiorgan failure was significantly more negative than that of group without multiorgan failure (− 1465.4 ± 154.9 ml vs. − 506.2 ± 23.0 ml, *P* < 0.001). Chest drainage (1483.6 ± 85.7 vs. 793.8 ± 17.8 ml, *P* < 0.001), and surgical duration (215.1 ± 6.1 vs. 179.7 ± 2.2 min, *P* < 0.001) of group with multiorgan failure were significantly more than those of group without multiorgan failure

Postoperatively, CVP decreased statistically significantly (*P* < 0.001), and LVEDD and LVEF improved statistically significantly (*P* < 0.001, *P* < 0.001; respectively (Table 4).

**Table 4:** Operative results (n = 826)

Variable	Preoperative	Post-operative	*P* value
CVP, mmHg	19.9 ± 0.2	11.7 ± 0.1	< 0.001
LVEDD, mm	41.8 ± 0.2	43.7 ± 0.2	< 0.001
LVEF, %	62.4 ± 0.3	64.4 ± 0.3	< 0.001
TI, cm^2^	1.8 ± 0.1	1.7 ± 0.1	0.210

Postoperatively, CVP decreased statistically significantly (*P* < 0.001), and LVEDD and LVEF improved statistically significantly (*P* < 0.001, *P* < 0.001; respectively)

The common early postoperative complications included acute renal injury (222/826, 26.9%), long-term intubation time > 48 h (393/826, 47.6%), and multiorgan failure (86/826, 10.4%).

### Analysis of risk factors of early mortality after pericardiectomy

Univariate analysis of potential risk factors of multiorgan failure showed that numerous factors are associated with multiorgan failure, including male (*P* < 0.001), age (*P* < 0.001), ICU retention time (*P* = 0.009), hospitalized time postoperative (*P* < 0.001), preoperative central venous pressure (*P* < 0.001), postoperative central venous pressure (*P* < 0.001), D0 fluid balance (*P* < 0.001), D2 fluid balance (*P* < 0.001), postoperative chest drainage (*P* < 0.001), surgical duration (*P* < 0.001), serum creatinine baseline (*P* < 0.001), serum creatinine 24 h after surgery (*P* < 0.001), serum creatinine 48 h after surgery (*P* < 0.001), fresh-frozen plasma (*P* = 0.001), blood lactate (*P* < 0.001), and tuberculosis pericarditis (*P* < 0.001).

When they were included in multivariate analysis models, multivariate analyses also showed that numerous factors are associated with multiorgan failure, including male (*P* < 0.001), age (*P* < 0.001), ICU retention time (*P* < 0.001), hospitalized time postoperative (*P* < 0.001), preoperative central venous pressure (*P* = 0.018), postoperative central venous pressure (*P* < 0.001), D0 fluid balance (*P* < 0.001), D2 fluid balance (*P* < 0.001), postoperative chest drainage (*P* = 0.029), surgical duration (*P* = 0.003), serum creatinine baseline (*P* = 0.002), serum creatinine 24 h after surgery (*P* < 0.001), serum creatinine 48 h after surgery (*P* < 0.001), blood lactate (*P* < 0.001), and tuberculosis pericarditis (*P* = 0.033) (Table 5).

**Table 5:** Analysis of risk factors of multiorgan failure after pericardiectomy

Model	OR	95% CI	*P* value
*Univariate analysis of risk factors of multiorgan failure after pericardiectomy*			
Male	0.358	0.228–0.563	< 0.001
Age	0.996	0.981–1.011	0.617
Weight before diuresis	0.964	0.941–0.988	0.003
Weight after diuresis	0.958	0.932–0.985	0.003
Height	0.907	0.882–0.943	< 0.001
BMI before diuresis	1.053	0.984–1.127	0.136
BMI after diuresis	1.081	1.003–1.165	0.042
Time between symptoms and surgery	1.026	1.018–1.035	< 0.001
Bleeding during operation	1.001	1.000–1.002	< 0.001
Thickness of pericardium	1.084	1.042–1.129	< 0.001
Intubation time	1.014	1.011–1.017	< 0.001
ICU retention time	1.249	1.194–1.306	< 0.001
Hospitalized time postoperative	1.044	1.026–1.062	< 0.001
Preoperative CVP	1.215	1.163–1.269	< 0.001
Postoperative CVP	1.174	1.098–1.254	< 0.001
Preoperative LVEDD	0.899	0.855–0.946	< 0.001
Postoperative LVEDD	0.695	0.614–0.788	< 0.001
D0 fluid balance	1.001	1.001–1.002	< 0.001
D2 fluid balance	0.999	0.998–0.999	< 0.001
D1 fluid balance	1.000	1.000–1.000	0.692
Serum creatinine baseline	1.002	0.996–1.009	0.427
Serum creatinine 24 h after surgery	1.041	1.032–1.051	< 0.001
Serum creatinine 48 h after surgery	1.046	1.038–1.054	< 0.001
Fresh-frozen plasma	1.001	1.001–1.002	< 0.001
Packed red cells	1.504	1.288–1.756	< 0.001
Surgical duration	1.012	1.009–1.016	< 0.001
Preoperative tricuspid regurgitation	0.964	0.876–1.061	0.456
Tuberculosis pericarditis	0.488	0.304–0.784	0.003
Blood lactate	1.891	1.696–2.109	< 0.001
Chest drainage	1.002	1.001–1.002	< 0.001
*Multivariate analysis of risk factors of multiorgan failure after pericardiectomy*			
Male	0.369	0.182–0.750	0.006
Weight before diuresis	0.984	0.899–1.077	0.730
Weight after diuresis	1.039	0.936–1.153	0.473
Intubation time	1.014	1.011–1.017	< 0.001
ICU retention time	1.571	1.373–1.799	< 0.001
Hospitalized time postoperative	0.888	0.848–0.929	< 0.001
D0 fluid balance	1.004	1.002–1.005	< 0.001
D2 fluid balance	0.999	0.998–0.999	< 0.001
Serum creatinine 24 h after surgery	0.976	0.954–0.999	0.042
Serum creatinine 48 h after surgery	1.092	1.067–1.117	< 0.001
Fresh-frozen plasma	1.003	1.002–1.004	< 0.001
Tuberculosis pericarditis	1.313	0.567–3.307	0.525
Surgical duration	1.010	1.005–1.014	< 0.001
Chest drainage	1.002	1.001–1.003	< 0.001
Preoperative CVP	1.751	1.435–2.138	< 0.001
Postoperative CVP	0.853	0.662–1.099	0.219
Blood lactate	9.273	3.784–22.72	< 0.001

Numerous factors are associated with multiorgan failure, including male (*P* < 0.001), age (*P* < 0.001), ICU retention time (*P* < 0.001), hospitalized time postoperative (*P* < 0.001), preoperative central venous pressure (*P* = 0.018), postoperative central venous pressure (*P* < 0.001), D0 fluid balance (*P* < 0.001), D2 fluid balance (*P* < 0.001), postoperative chest drainage (*P* = 0.029), surgical duration (*P* = 0.003), serum creatinine baseline (*P* = 0.002), serum creatinine 24 h after surgery (*P* < 0.001), serum creatinine 48 h after surgery (*P* < 0.001), blood lactate (*P* < 0.001), and tuberculosis pericarditis (*P* = 0.033)

### Histopathologic study results

Histopathologic studies of pericardium tissue from every patient were done. The diagnosis of tuberculosis was confirmed on the basis of clinical findings and histopathologic features, including the presence of typical granuloma and caseous necrosis, acid-fast bacilli in Ziel-Nelson tissue staining, and bacteriologic studies using the polymerase chain reaction (PCR) test on the pericardial fluid or tissue for evidence of mycobacterium tuberculosis.

In this series from Guangxi, China, characteristic histopathologic features of tuberculosis (434/826, 52.5%) of pericardium were the most common histopathologic findings, and 260 patients (392/826, 47.5%) had the histopathologic findings of chronic nonspecific inflammatory changes.

### Follow-up results

**Fig. 1:**
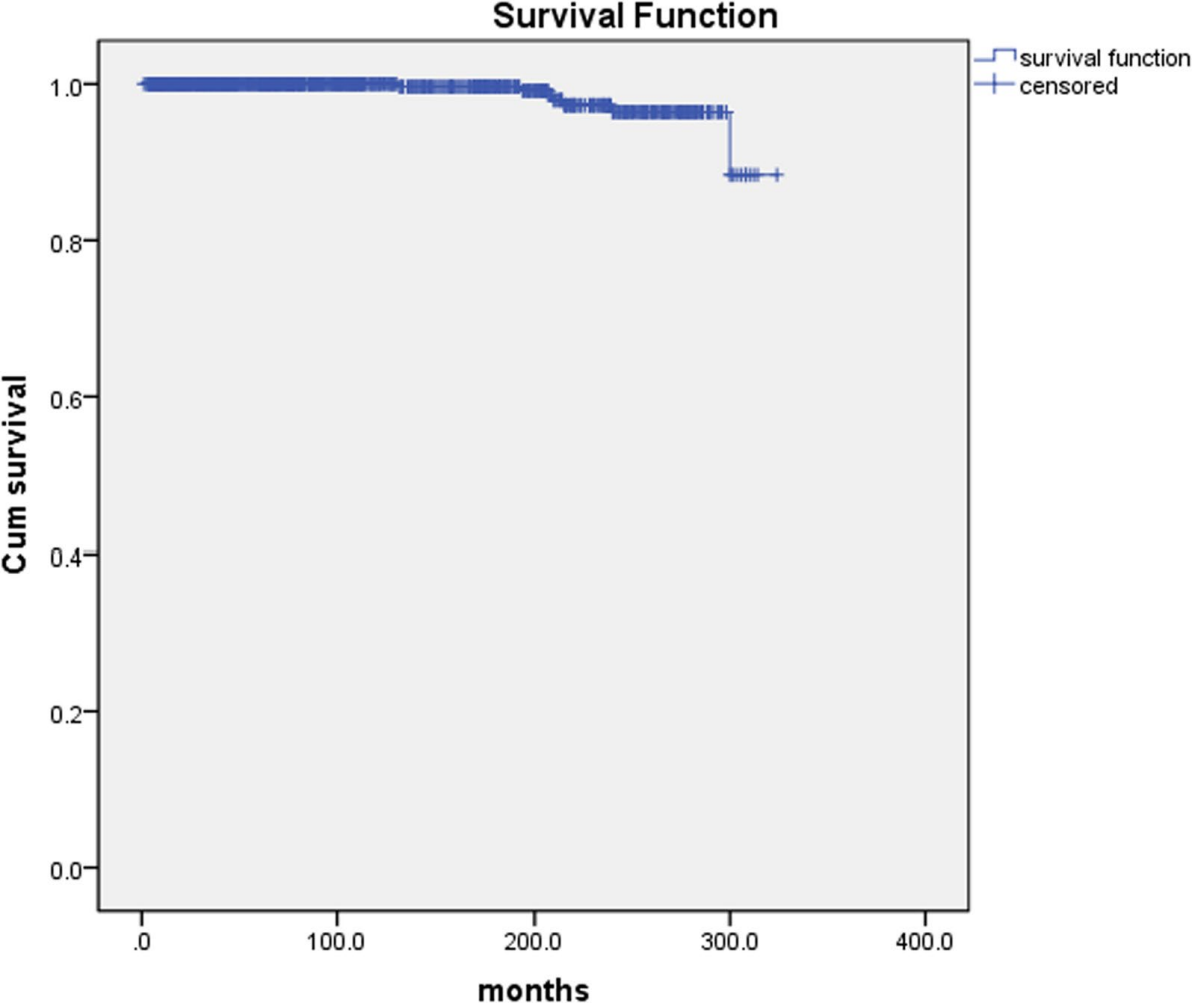
Kaplan–Meier curve for survival. 760 survivors were discharged from hospital and 684 patients were monitored to the end date of the study and the follow-up was 90.0% (684/760) completed. The mean duration of follow-up was 126.4 ± 3.5 months (range 1 to 342), 7 late deaths (7/684, 1.0%) occurred

760 survivors were discharged from hospital and 684 patients were monitored to the end date of the study and the follow-up was 90.0% (684/760) completed. The mean duration of follow-up was 126.4 ± 3.5 months (range 1 to 342), 7 late deaths (7/684, 1.0%) occurred 131, 193, 208, 210, 215, 240, and 300 months after being discharged from our hospital. 3 died of heart failure, 1 of cerebral hemorrhage, and 3 of unknown reason. The latest data of follow-up showed that 656 survivors were in NYHA class I (656/684, 95.9%) and 21 in class II (21/684, 3.1%) (Fig. 1).

## Discussion

Constrictive pericarditis arises as a result of the fibrous thickening of the pericardium due to chronic inflammatory changes from various injuries. Increased pulmonary and systemic venous pressures manifest clinical features of left and right heart failure. Right-sided heart failure symptoms predominate over left-sided heart failure symptoms due to the equalization of pressures [1–3].

### Causes of multiorgan failure following pericardiectomy

In our study, the causes of operative deaths were multiorgan failure, including cardiogenic shock + AKI + ventricular fibrillation (13/66), cardiogenic shock + AKI (35/66), cardiogenic shock + AKI + hepatic failure + septicemia (8/66), cardiogenic shock + AKI + respiratory failure (10/66).

### Incomplete pericardial dissection is associated with multiorgan failure following pericardiectomy

The causes of low cardiac output syndrome are related to the incomplete resection of thickened pericardium, unsatisfactory relief of left ventricular compression, excessive ventricular dilatation after pericardial dissection, myocardial weakness, and heart failure [8]. In our study, univariate and multivariate analyses showed that postoperative LVEDD (*P* < 0.001) is associated with multiorgan failure (Table 5), indicating that the incomplete resection of thickened pericardium and unsatisfactory relief of left ventricular compression are associated with multiorgan failure following pericardiectomy.

We removed the pericardium from phrenic nerve to phrenic nerve without CPB as the procedure of choice. However, this often results in insufficient removal of pericardium to relieve the constriction, especially in cases of complete encirclement of the heart, most frequently around the base by a heavily thickened calcified ring. In these situations, the post-lateral and inferior wall pericardial thickening that are sometimes associated with severe cardiac compression are left behind. Therefore, in severe constrictive pericarditis as these, the textbook approach of phrenic-to-phrenic removal will often be not nearly enough to relieve the constriction. It is perhaps for this reason that there was such a high percentage of patients experiencing LCOS after pericardiectomy.

Therefore, complete pericardiectomy (phrenic to phrenic removal and removal of the post-lateral and inferior wall pericardial thickening) on CPB for complete relief of the constriction of the heart should be the routine. We have done 3 cases isolated pericardiectomy under CPB in the present study. The complete pericardiectomy under CPB has the possibility of the increased bleeding compared with off pump pericardiectomy.

### Improvement of surgical techniques

Bleeding during operation (518.6 ± 28.4 vs. 385.0 ± 11.9 ml, *P* < 0.001), chest drainage (1468.6 ± 68.5 vs. 789.6 ± 18.5 ml, *P* < 0.001), and surgical duration (230.0 ± 5.7 vs. 174.1 ± 2.1 min, *P* < 0.001) of group with multiorgan failure were significantly more than those of group without multiorgan failure. Univariate and multivariate analyses showed that bleeding during operation (*P* = 0.005), chest drainage (*P* < 0.001), and surgical duration (*P* < 0.001) are associated with multiorgan failure (Table 5). Improvement of surgical techniques can decrease bleeding during operation, chest drainage, and surgical duration [9].

### Fluid balance on operation day and fluid balance postoperative day D2 are associated with multiorgan failure following pericardiectomy

Fluid balance on operation day (D0) of group with multiorgan failure were significantly less negative than that of group without multiorgan failure (− 640.7 ± 52.0 ml vs. − 1223.9 ± 32.6 ml, *P* < 0.001). While fluid balance postoperative day D2 of group with multiorgan failure was significantly more negative than that of group without multiorgan failure (− 1176.1 ± 154.9 ml vs. − 478.9 ± 20.9 ml, *P* < 0.001). D0 fluid balance (*P* < 0.001), D2 fluid balance (*P* < 0.001), Univariate and multivariate analyses showed that D0 fluid balance (*P* < 0.001) and D2 fluid balance (*P* < 0.001) are associated with multiorgan failure (Table 5). Fluid overload must be avoided during the post-operative period in cardiac surgery. A negative fluid balance is preferable after cardiac surgery to avoid severe complications such as lung edema. A positive fluid balance after cardiac surgery has been associated with increased mortality [10]. Our study showed that fluid balance on operation day (D0) and postoperative day D1 should be negative enough to optimize the preload of the heart.

### Early diagnosis and treatment of constrictive pericarditis

In our study, time between symptoms and surgery (22.9 ± 4.1 vs. 6.7 ± 0.5 month, *P* < 0.001), thickness of pericardium (22.7 ± 0.6 vs. 19.8 ± 0.2 mm, *P* < 0.001), preoperative CVP (25.0 ± 0.4 vs. 19.3 ± 0.2 mmHg, *P* < 0.001) in group with multiorgan failure were significantly higher than those in group without multiorgan failure (Table 2). Univariate and multivariate analyses showed that factors including time between symptoms and surgery (*P* < 0.001), thickness of pericardium (*P* < 0.001), preoperative CVP (*P* < 0.001) are associated with multiorgan failure (Table 5). Therefore, early diagnosis and treatment of constrictive pericarditis are important. Early surgical intervention is advocated, as constrictive pericarditis is a progressive disease, and patients with a poor preoperative functional class are at the highest risk for perioperative death. Pericardiectomy is indicated once the diagnosis of constrictive pericarditis is made. Systematic antituberculosis drugs should be given to patients with constrictive pericarditis caused by tuberculous bacteria. Surgery should be performed after body temperature, erythrocyte sedimentation rate, and general nutritional status are normal or relatively stable and before cardiogenic cachexia and severe live function injury occur [11, 12].

Low cardiac output syndrome is fundamental in etiology of multiorgan failure following pericardiectomy. Incomplete pericardial dissection is associated with low cardiac output syndrome. Low cardiac output syndrome, AKI, ARDS, and hepatic failure can affect each other.

#### Study limitations

Limitations of the present study include its retrospective design. There may be a selection bias because of the retrospective nature of the study.

## Conclusions

In our study, incomplete pericardial dissection, fluid overload, delayed diagnosis and treatment are associated with multiorgan failure following pericardiectomy. Further studies toned to be conducted with a larger sample size to confirm our study results.

## Data Availability

The datasets generated and/or analyzed during the current study are available from the corresponding author on reasonable request.

## Abbreviations

LCOS: Low cardiac output syndrome
LVEDD: Left ventricular end diastolic dimension
CT: Computed tomograph
CPB: Cardiopulmonary bypass
CO: Cardiac output
CI: Cardiac index
ICU: Intensive care unit
MOF: Multiple organ failure
ECMO: Extracorporeal membrane oxygenation
CVP: Central venous pressure
LVEDD: Left ventricular end diastolic dimension
LVEF: Left ventricular ejection fractions
ICU: Intensive care unit
CPB: Cardiopulmonary bypass
AKI: Acute renal injury
AKIN: Acute kidney injury network
BMI (body mass index): Weight/(height^2^) (kg/m^2^)
